# Supplementary material for: Cdc14 phosphatase contributes to cell wall integrity and pathogenesis in Candida albicans
Source: Front Microbiol. 2023 Feb 16;14:1129155. doi: 10.3389/fmicb.2023.1129155 (PMC9977832; doi:10.3389/fmicb.2023.1129155)
Supplement: Supplementary file 1 [file Data_Sheet_1.PDF]

SUPPLEMENTARY TABLES

Table S1. Plasmids

Species	Name	Backbone	Marker	Expressed Protein	Source
E. coli					
	pHLP603	pDEST17	Ampicillin	6xHis-ScCdc14(1-551)	This study
	pHLP695	pDEST17	Ampicillin	6xHis-ScCdc14(1-449)	This study
	pHLP690	pDEST17	Ampicillin	6xHis-ScCdc14(1-374)	This study
	pHLP621	pDEST17	Ampicillin	6xHis-ScCdc14(1-449^{QARA})	This study
	pHLP622	pDEST17	Ampicillin	6xHis-ScCdc14(1-449^{APRK})	This study
	pHLP683	pDEST17	Ampicillin	6xHis-CaCdc14(1-427)	This study
	pHLP687	pDEST17	Ampicillin	6xHis-CaCdc14(1-427^{AARA})	This study
		**Origin**			
S. cerevisiae					
	pRS314-*CDC14*	CEN/ARS	*TRP1*	ScCdc14	(1)
	pRS314-*cdc14*^{hm}	CEN/ARS	*TRP1*	ScCdc14^{QARA}	This study
C. albicans					
	pJC347	Integrating	*URA3*	CaCdc14-3xHA	This study
	pHLP661	Integrating	*URA3*	CaCdc14^{AARA}-3xHA	This study

Table S2. Yeast strains

	Strain Name	Relevant Genotype	Source
S. cerevisiae			
	W303	*MATα ade2-1 can1-100 his3-11,15 leu2-3,112 trp1-1 ura3-1*	
	YKA1038	W303 *MATα cdc14*^{hm}	This study
	HCY109	W303 *MATa cdc14::HIS3* pRS316-*CDC14*	(1)
	M609	W303 *MATa cdc14-3*	(2)
	hog1Δ	BY4741 *MATa hog1::*KanMX4	Horizon Discovery
	yap1Δ	BY4741 *MATa yap1::*KanMX4	Horizon Discovery
	LSY2244-90B	*MATα mus81::*KanMX6 *yen1::HIS3 his3::*HphMX4	(3)
S. pombe			
	KLG248	ura4-D18 ade6-216 leu1-32 h-	(4)
	KLG3381	Clp1::ura4 ura4-D18 ade6-216 leu1-32 h-	(4)
C. albicans			
	CAI4	*ura3::imm434/ura3::imm434*	(5)
	JC8	CAI4 *cdc14::hisG/cdc14::hisG*	(6)
	JC2712	CAI4 *NEUT5L::URA3*	This study
	JC2711	CAI4 *NEUT5L::URA3 cdc14::hisG/cdc14::hisG*	This study
	JC2721	CAI4 *CDC14-3HA:URA3/cdc14::hisG*	This study
	HCAL102	CAI4 *cdc14*^{hm}*-3HA:URA3/cdc14::hisG*	This study

Table S3. Primers for qRT-PCR

Primer Name	Sequence (5'-3')
S. cerevisiae	
ScACT1-001F	TGGATTCCGGTGATGGTGTT
ScACT1-002R	TCAAAATGGCGTGAGGTAGAGA
ScFKS2-001F	GATGGGACTGGTGACGGTAATTA
ScFKS2-002R	TCATCGTACGCACTTTGATCCT
C. albicans	
CaRDN18-001F	GCTGATGACTTGCGCTTACTA
CaRDN18-002R	GAGAGGTCTGGGAAATCTTGTG
CaECM331-001F	GTTGGTCTCATTACCTTCCT
CaECM331-002R	CCAACCCGTTCAAATTAGTC
CaPGA13-001F	CCACCACTGCTGAACAACCA
CaPGA13-002R	GCAGGAGTAGTAACCACAGATTCAATA
CaPGA31-001F	TGGTTCCTCCACTGTCAGACAA
CaPGA31-002R	ACCACTACCACCCAATTGAAGAA
CaCRH11-001F	TGGTCCGTTGACGGTAGTGTT
CaCRH11-002R	TTGTGGGAAACCTTGTGCATT
CaENG1-001F	GGGCAACTGGTTTGTTCGTC
CaENG1-002R	ACCACCTCTTGCTTCCATCG
CaDSE1-001F	AACGAAGATCAACGTCAAAGTGATAA
CaDSE1-002R	AACTTCATTTTCAACATCAGTCACAAC
CaURA3-001F	GCTCATGGTGTCACTGGGAAT
CaURA3-002R	GGTTGGTGGTGGTTTCTTTAGC

SUPPLEMENTARY FIGURE LEGENDS

Figure S1. Comparison of *URA3* marker gene expression in *C. albicans* strains. Relative mRNA levels of the *URA3* marker gene used to create the indicated strains via integration at either *NEUT5L* or *CDC14* loci were measure by qRT-PCR from samples of mid-log phase liquid YPD cultures. Transcript levels were normalized to *RDN18* and set relative to the wild-type sample. Data are averages of 3 biological replicates and error bars are standard deviations. Unpaired T-tests assuming equal variance were used to compare the average expression to that of the wild-type strain. "ns" = not significant ($p >$ 0.05).

Figure S2. Purification and characterization of ScCdc14 enzymes. (**A**) 2 µg of ScCdc14 enzyme variants purified from *E. coli* by nickel affinity chromatography were evaluated by SDS-PAGE with Coomassie blue staining. M = molecular weight markers. Lane labels indicate expressed amino acid regions and any amino acid substitutions made in the QPRK motif. (**B**) Relative rates of ScCdc14(1-449^{QARA})-catalyzed dephosphorylation of the phosphopeptide sequences shown in the table at left were measured under identical conditions at 100 µM each substrate. Peptide 1 is the same near-optimal substrate sequence used in Fig 1C and 1F and the other peptides contain substitutions at positions known to influence substrate recognition by ScCdc14. Activity sharply decreased with changes to phosphoamino acid (pS, pT, pY) and the +1 and +3 positions relative to pS, similar to wild-type Cdc14 enzymes (7,8).

Figure S3. Sequence alignment of fungal and animal Cdc14 orthologs. Alignment of selected fungal (light blue) and animal (red) *CDC14* homolog sequences was generated using Clustal Omega with default settings. The location of the fungal-specific QPRK motif is indicated by yellow highlighting and conserved residues in the active site region responsible for catalysis and substrate recognition are labeled.

Figure S4. Characterization of *cdc14^{hm}* allele in *S. cerevisiae*. (**A**) Microplate growth assay in liquid YPAD performed at 30°C. (**B-E**) *S. cerevisiae* strains were serially diluted and spotted on YPAD agar plates containing indicated concentrations of (**B**) hydroxyurea (HU), (**C**) sodium chloride (NaCl), (**D**)

hydrogen peroxide (H_2O_2), and (**E**) Congo red (CR). "# 1-3" indicates independent isolates of *cdc14*^{hm}/*Δ* strain. *yen1Δ mus81Δ*, *yap1Δ*, and *hog1Δ* strains are positive controls for sensitivity to HU, NaCl, and H_2O_2, respectively. (**F**) The same spotting assays on YPAD plates grown at the indicated temperatures. (**G**) Spotting assays were performed with a *cdc14Δ* plasmid shuffle strain kept alive by CEN plasmids expressing either *cdc14*^{hm} or wild-type *CDC14* alleles expressed from the natural *CDC14* promoter. (**H**) Spotting assays with *cdc14*^{hm} with and without a CEN plasmid expressing wild-type *CDC14* from its natural promoter, compared to wild-type control. All plates were incubated at 30°C (unless otherwise specified) for 72 hours. Wild-type refers to W303 parent strain. Images are representative of three biological replicates. (**I**) Gene expression (qRT-PCR) analysis of *FKS2* in Wild-type and *cdc14*^{hm}. *FKS2* transcript levels were normalized to *ACT1* and set relative to wild-type. Error bars represent standard deviation of three biological replicates each with three technical repeats. T-test p value = 0.064.

Figure S5. Characterization of *cdc14*^{hm} allele in *C. albicans.* (**A**) 2 µg of truncated (residues 1-427) CaCdc14 enzymes (wild-type and QPRK→AARA mutant) purified from *E. coli* by nickel affinity chromatography were analyzed by SDS-PAGE with Coomassie blue staining. M = molecular weight marker. (**B**) Comparison of reaction velocities of CaCdc14(1-427) and CaCdc14(1-427^{AARA}) towards a collection of peptides with substitutions at positions important for optimal Cdc14 substrates. Activity is sharply reduced by changes to phosphoamino acid (pS, pT, pY) or the +1 or +3 positions relative to pS, similar to wild-type Cdc14 enzymes from diverse species (8). (**C**) Anti-HA immunoblotting analysis of *C. albicans cdc14Δ/Δ* complemented with wild-type *CDC14-3xHA* and *cdc14*^{hm}*-3xHA* integration cassettes in the absence and presence of 50 ng/ml micafungin (MF). Anti-PSTAIR is a loading control. "# 1-3" refers to individual isolates of *cdc14*^{hm}/*Δ* strain. (**D**) Growth of the indicated *C. albicans* strains in YPD liquid cultures was monitored by absorbance at 600 nm. Samples were sonicated and diluted to $A_{600} < 1.5$ prior to measurement and the undiluted absorbance equivalents plotted. Average values with standard deviation error bars from 3 independent cultures per strain are plotted. Fit curves were generated in GraphPad Prism using the Gompertz growth function. (**E**) *C. albicans* strains were serially diluted and

spotted on YPD plates containing the indicated amounts of sodium chloride (NaCl), methyl methanesulfonate (MMS), or hydrogen peroxide (H_2O_2). The concentration of stress agents was the maximum tolerated by the wild-type strain without significant reduction in viability or growth rate. All plates were incubated at 30°C for 72 hours. Images are representative of three independent trials.

Figure S6. Relative mRNA levels of selected cell wall stress-responsive genes in mid-log phase cultures of *C. albicans* strains were measured by qRT-PCR. Transcript levels were normalized to *RDN18* and set relative to the wild-type sample. Data are averages of 6 biological trials and error bars are standard deviations. Unpaired T-tests assuming equal variance were used to compare the average expression to that of the wild-type strain (* = $p < 0.05$).

Figure S7. Hyphal development is impaired in *cdc14^{hm}*/Δ. (**A-B**) *C. albicans* strains were streaked out on YPD + 10% fetal bovine serum (FBS) and allowed to grow at 37°C for 5 days. Colonies were imaged before and after washing of the plate surface with water. Embedded cells were excised in small agar plugs and imaged by DIC microscopy using a 40x objective. (**C**) *C. albicans* cells grow in YPD + 10% FBS liquid medium at 37 °C for 2 hours were similarly imaged by DIC microscopy. The length of hyphal projections, defined as cell bodies with a length at least twice the width, were measured using ImageJ (minimum 70 per strain). Statistical significance was determined with unpaired T-tests assuming equal variance comparing each strain to wild-type (* = $p < 0.05$; ** = $p < 0.01$).

Figure S8. Establishment of mouse model of invasive candidiasis. Immunosuppressed BALB/c mice were infected with indicated amounts of either wild-type **(A)** or *cdc14Δ/Δ* **(B)** *C. albicans* cells or mock-infected to determine the minimum wild-type dose required for complete mortality and assess initial *CDC14* requirement for virulence. *p* values were determined using a log-rank (Mantel-Cox) test comparing the survival distribution of mice infected with wild-type vs *cdc14Δ/Δ* strains at each fungal cell dose. ns = not significant ($p > 0.05$). **C)** Representative images (200x) of liver and lung tissue sections from an uninfected mouse from experiment in Fig. 7B.

SUPPLEMENTARY REFERENCES

1. Taylor GS, Liu Y, Baskerville C, Charbonneau H. The activity of Cdc14p, an oligomeric dual specificity protein phosphatase from Saccharomyces cerevisiae, is required for cell cycle progression. J Biol Chem. 1997 Sep 19;272(38):24054–63.

2. Miller CT, Gabrielse C, Chen YC, Weinreich M. Cdc7p-Dbf4p Regulates Mitotic Exit by Inhibiting Polo Kinase. PLOS Genet. 2009 May 29;5(5):e1000498.

3. Ho CK, Mazon G, Lam AF, Symington LS. Mus81 and Yen1 promote reciprocal exchange during mitotic recombination to maintain genome integrity in budding yeast. Mol Cell. 2010 Dec 22;40(6):988–1000.

4. Broadus MR, Gould KL. Multiple protein kinases influence the redistribution of fission yeast Clp1/Cdc14 phosphatase upon genotoxic stress. Mol Biol Cell. 2012 Oct;23(20):4118–28.

5. Fonzi WA, Irwin MY. Isogenic strain construction and gene mapping in Candida albicans. Genetics. 1993 Jul 1;134(3):717–28.

6. Clemente-Blanco A, Gonzalez-Novo A, Machin F, Caballero-Lima D, Aragon L, Sanchez M, et al. The Cdc14p phosphatase affects late cell-cycle events and morphogenesis in Candida albicans. J Cell Sci. 2006 Mar 15;119(Pt 6):1130–43.

7. Bremmer SC, Hall H, Martinez JS, Eissler CL, Hinrichsen TH, Rossie S, et al. Cdc14 phosphatases preferentially dephosphorylate a subset of cyclin-dependent kinase (Cdk) sites containing phosphoserine. J Biol Chem. 2012 Jan 13;287(3):1662–9.

8. DeMarco AG, Milholland KL, Pendleton AL, Whitney JJ, Zhu P, Wesenberg DT, et al. Conservation of Cdc14 phosphatase specificity in plant fungal pathogens: implications for antifungal development. Sci Rep. 2020 Dec;10(1):12073.

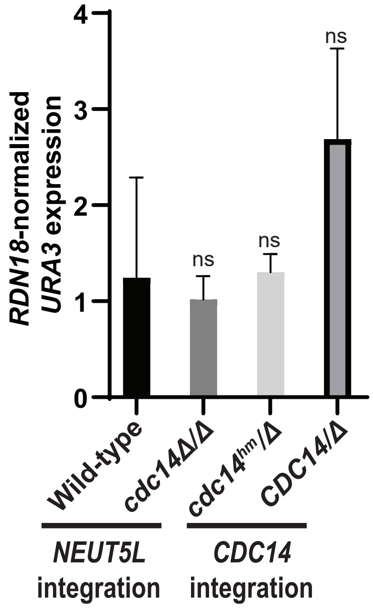

RDN18-normalized
URA3 expression
4
3
2
1
0
ns
ns
ns
Wild-type
cdc14Δ/Δ
cdc14hm/Δ
CDC14/Δ
NEUT5L
integration
CDC14
integration

Figure S1

A

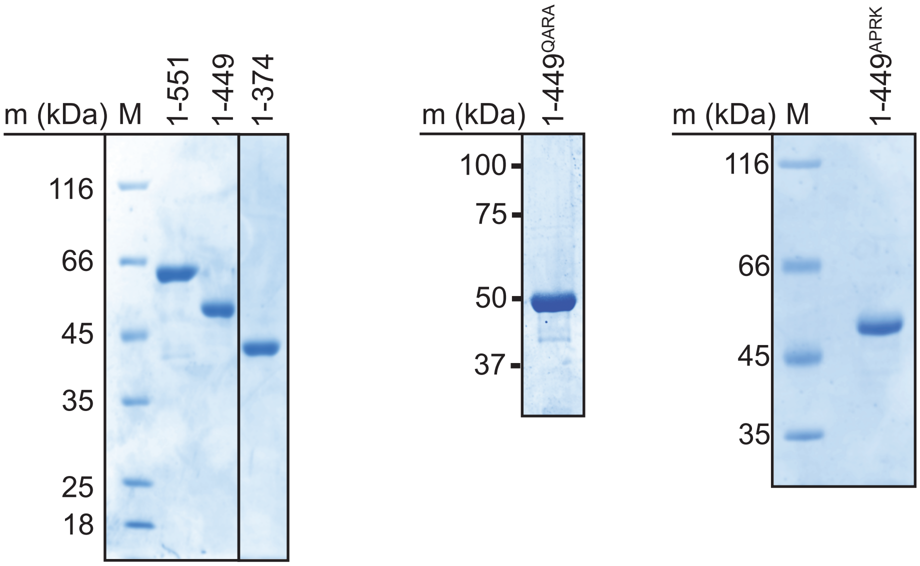

B

Name	Peptide Sequence
pS	HT(pS)PIKSIG
pT	HT(pT)PIKSIG
pY	HT(pY)PIKSIG
+1A	HT(pS)AIKSIG
+2K	HT(pS)PKKSIG
+2R	HT(pS)PRKSIG
+3A	HT(pS)PIASIG
+3R	HT(pS)PIRSIG
+4K	HT(pS)PIKKIG
+4R	HT(pS)PIKRIG
+2K,+3A	HT(pS)PKASIG
+3A,+4K	HT(pS)PIAKIG

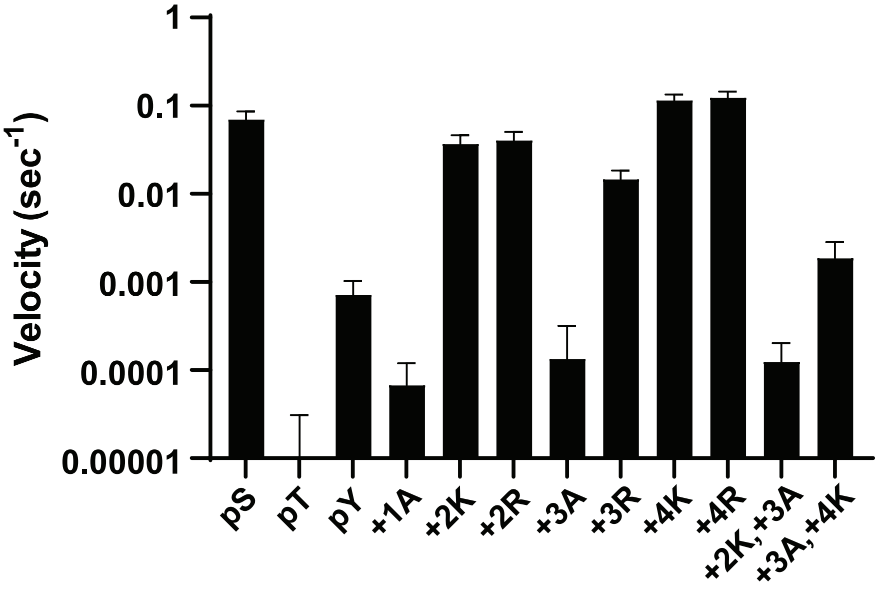

Figure S2

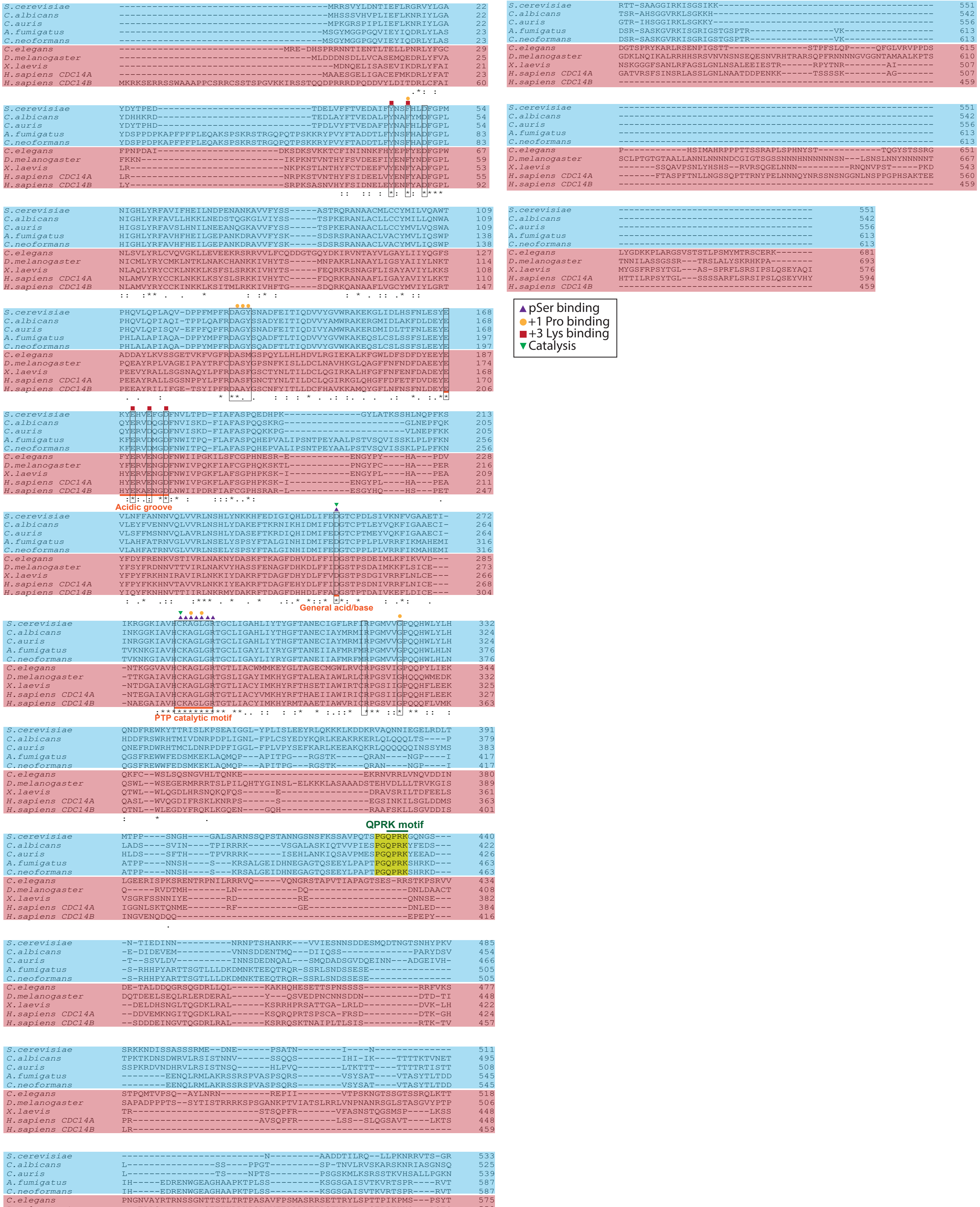
S.cerevisiae
C.albicans
C.auris
A.fumigatus
C.neoformans
C.elegans
D.melanogaster
X.laevis
H.sapiens CDC14A
H.sapiens CDC14B
pSer binding
+1 Pro binding
+3 Lys binding
Catalysis
Acidic groove
General acid/base
PTP catalytic motif
QPRK motif

Figure S3

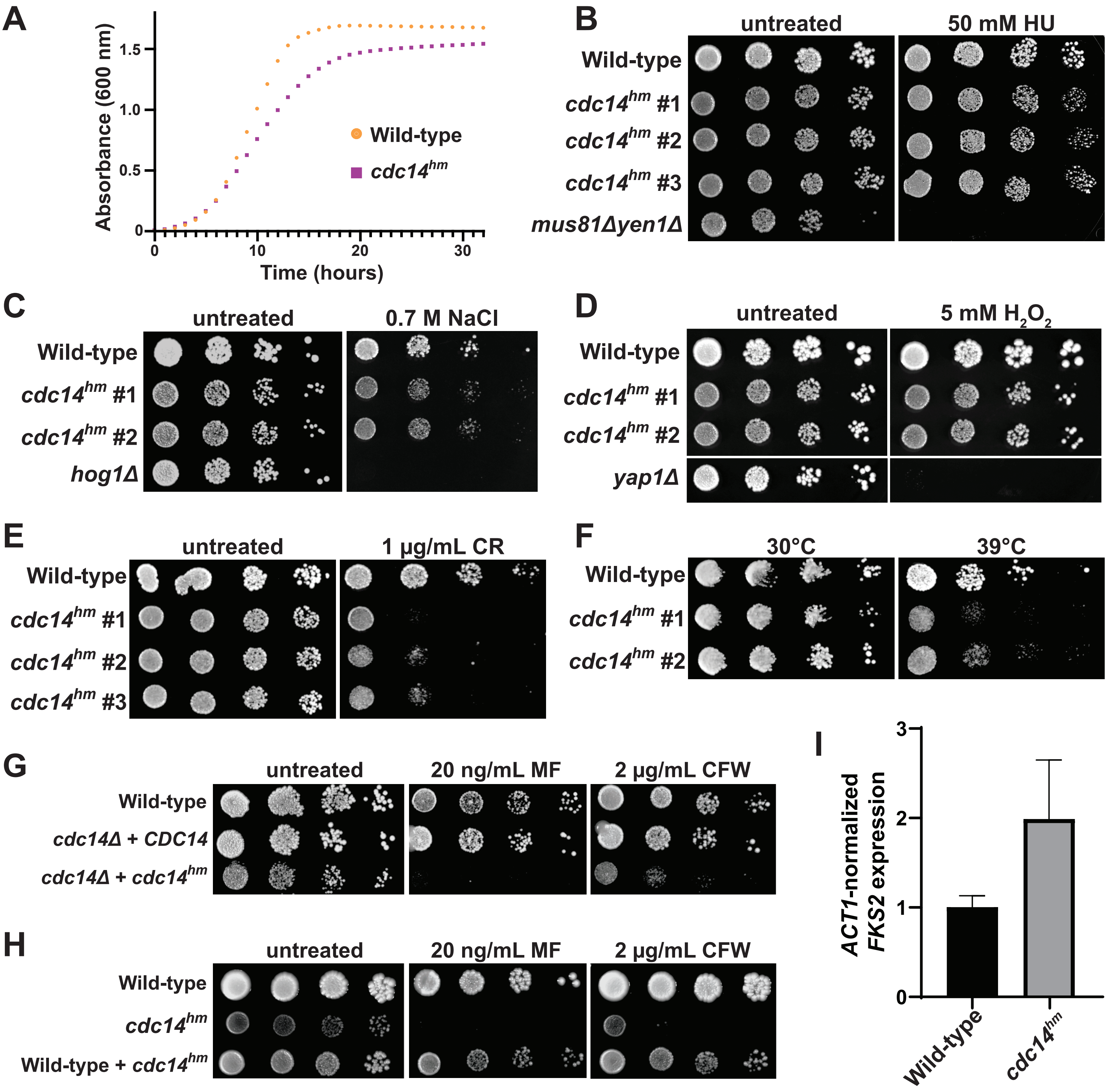
A
Absorbance (600 nm)
1.5
1.0
0.5
0
0
10
20
30
Time (hours)
Wild-type
cdc14hm
B
untreated
50 mM HU
Wild-type
cdc14hm #1
cdc14hm #2
cdc14hm #3
mus81Δyen1Δ
C
untreated
0.7 M NaCl
Wild-type
cdc14hm #1
cdc14hm #2
hog1Δ
D
untreated
5 mM H2O2
Wild-type
cdc14hm #1
cdc14hm #2
yap1Δ
E
untreated
1 µg/mL CR
Wild-type
cdc14hm #1
cdc14hm #2
cdc14hm #3
F
30°C
39°C
Wild-type
cdc14hm #1
cdc14hm #2
G
untreated
20 ng/mL MF
2 µg/mL CFW
Wild-type
cdc14Δ + CDC14
cdc14Δ + cdc14hm
H
untreated
20 ng/mL MF
2 µg/mL CFW
Wild-type
cdc14hm
Wild-type + cdc14hm
I
ACT1-normalized FKS2 expression
3
2
1
0
Wild-type
cdc14hm

Figure S4

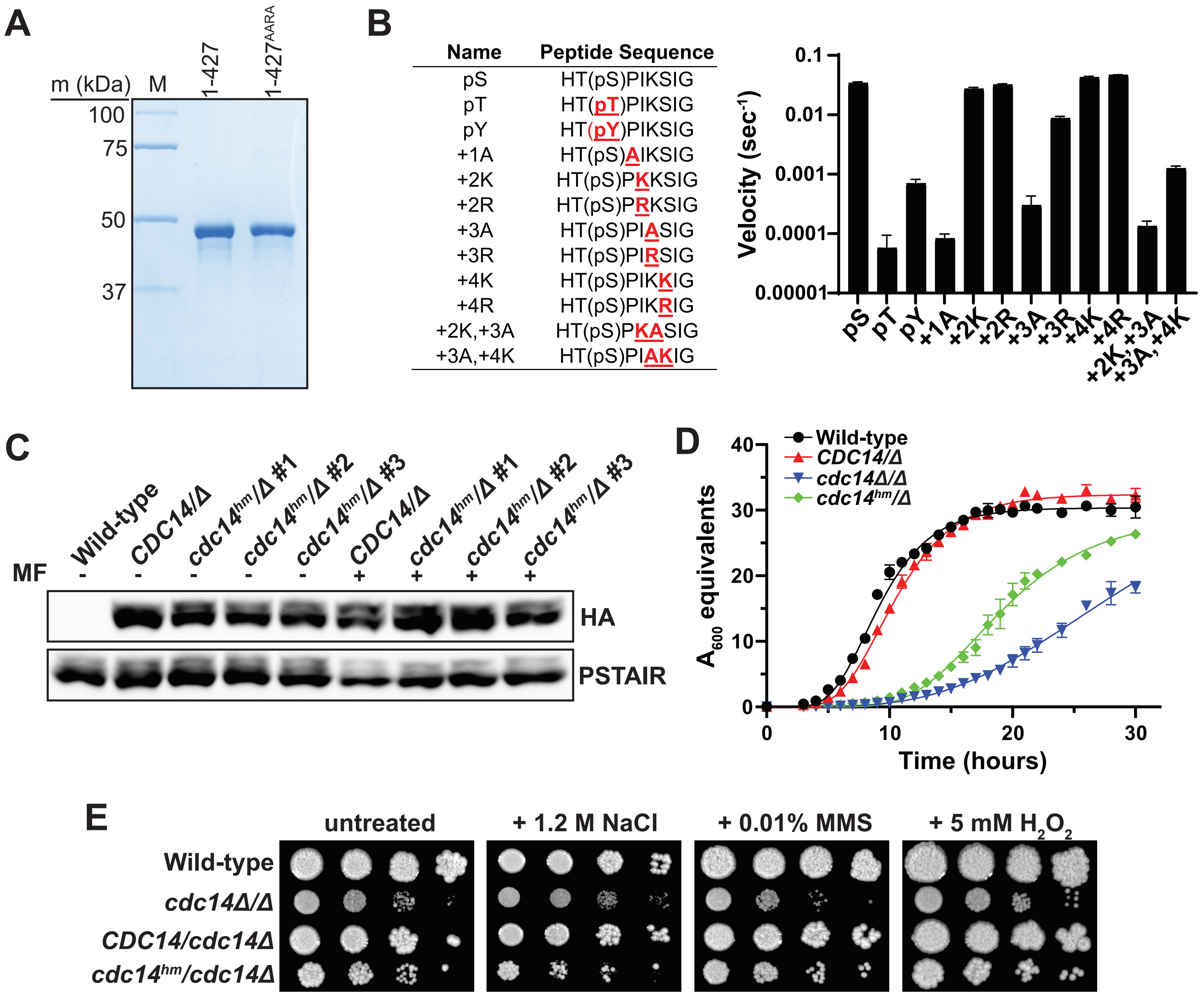
A
m (kDa)
M
1-427
1-427AARA
100
75
50
37
B
Name
Peptide Sequence
pS HT(pS)PIKSIG
pT HT(pT)PIKSIG
pY HT(pY)PIKSIG
+1A HT(pS)AIKSIG
+2K HT(pS)PKKSIG
+2R HT(pS)PRKSIG
+3A HT(pS)PIASIG
+3R HT(pS)PIRSIG
+4K HT(pS)PIKKIG
+4R HT(pS)PIKRIG
+2K,+3A HT(pS)PKASIG
+3A,+4K HT(pS)PIAKIG
Velocity (sec-1)
0.1
0.01
0.001
0.0001
0.00001
pS
pT
pY
+1A
+2K
+2R
+3A
+3R
+4K
+4R
+2K,+3A
+3A,+4K
C
MF
Wild-type
CDC14/Δ
cdc14hm/Δ #1
cdc14hm/Δ #2
cdc14hm/Δ #3
CDC14/Δ
cdc14hm/Δ #1
cdc14hm/Δ #2
cdc14hm/Δ #3
- - - - - + + + +
HA
PSTAIR
D
Wild-type
CDC14/Δ
cdc14Δ/Δ
cdc14hm/Δ
A600 equivalents
40
30
20
10
0
0
10
20
30
Time (hours)
E
untreated
+ 1.2 M NaCl
+ 0.01% MMS
+ 5 mM H2O2
Wild-type
cdc14Δ/Δ
CDC14/cdc14Δ
cdc14hm/cdc14Δ

Figure S5

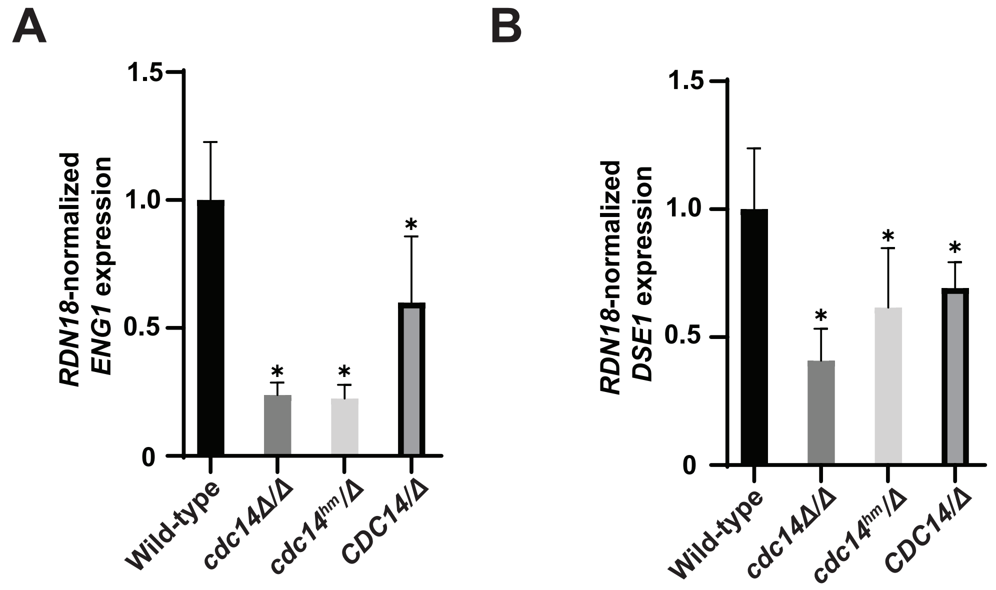
A
B
RDN18-normalized ENG1 expression
RDN18-normalized DSE1 expression
1.5
1.0
0.5
0
*
Wild-type
cdc14Δ/Δ
cdc14hm/Δ
CDC14/Δ

Figure S6

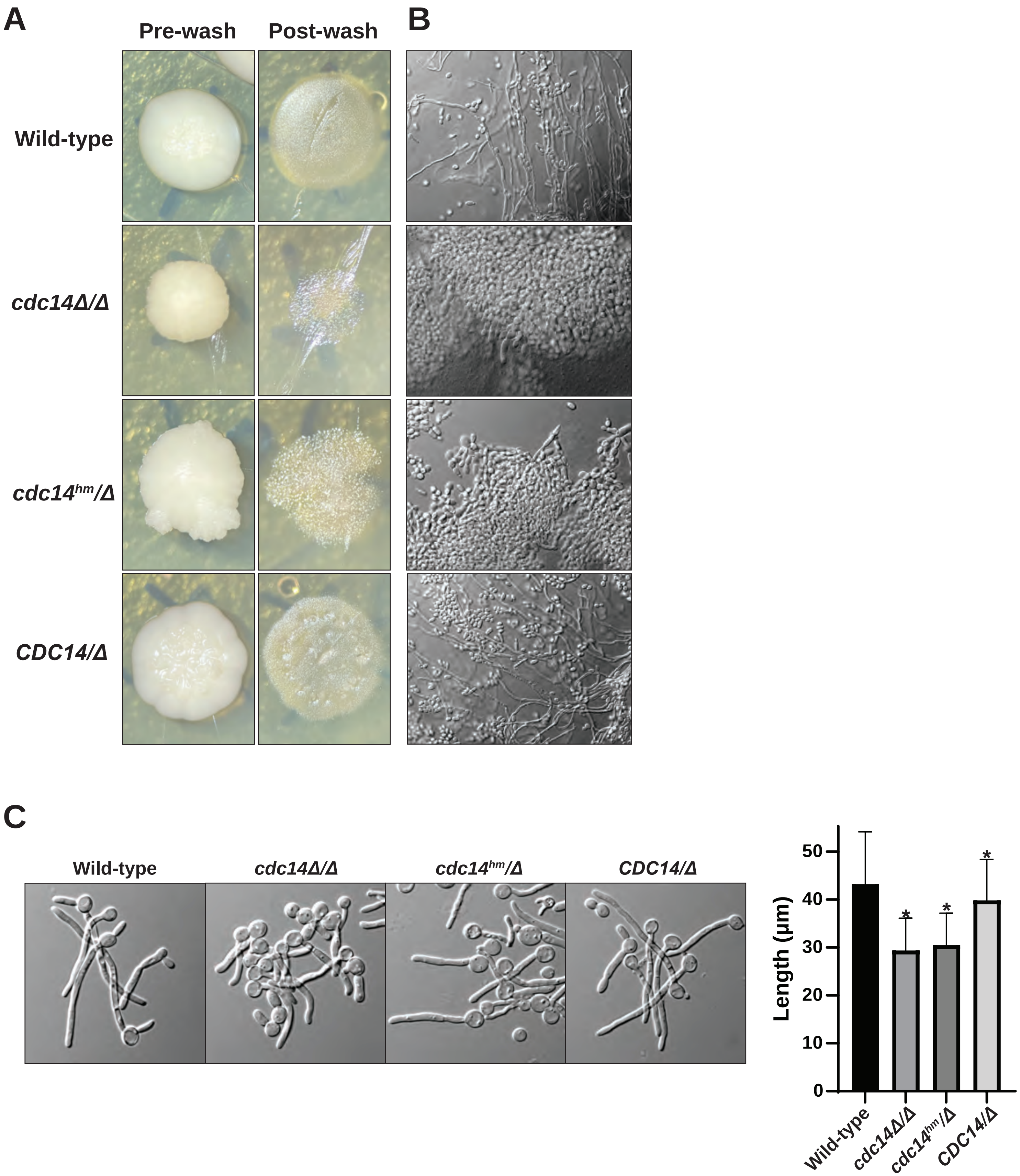
A
Pre-wash
Post-wash
B
Wild-type
cdc14Δ/Δ
cdc14hm/Δ
CDC14/Δ
C
Wild-type
cdc14Δ/Δ
cdc14hm/Δ
CDC14/Δ
Length (μm)
0
10
20
30
40
50
*
Wild-type
cdc14Δ/Δ
cdc14hm/Δ
CDC14/Δ

Figure S7

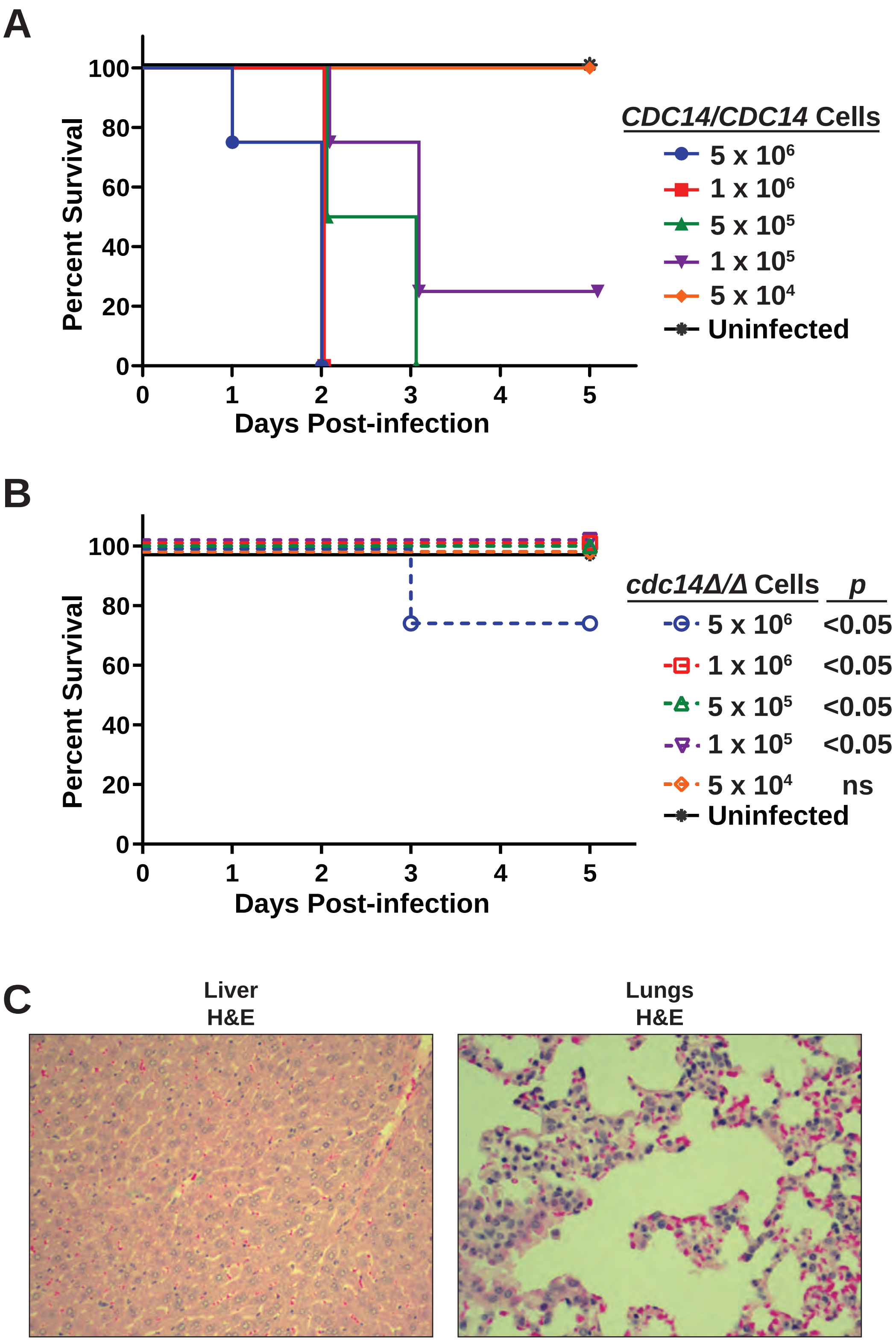
A
Percent Survival
100
80
60
40
20
0
0
1
2
3
4
5
Days Post-infection
CDC14/CDC14 Cells
5 x 10^6
1 x 10^6
5 x 10^5
1 x 10^5
5 x 10^4
Uninfected
B
Percent Survival
100
80
60
40
20
0
0
1
2
3
4
5
Days Post-infection
cdc14Δ/Δ Cells
p
5 x 10^6 <0.05
1 x 10^6 <0.05
5 x 10^5 <0.05
1 x 10^5 <0.05
5 x 10^4 ns
Uninfected
C
Liver
H&E
Lungs
H&E

Figure S8
